# Psychedelic literary studies and the poetics of disruption

**DOI:** 10.3389/fpsyg.2023.1155908

**Published:** 2023-06-09

**Authors:** Neşe Devenot, George Erving

**Affiliations:** ^1^Institute for Research in Sensing, University of Cincinnati, Cincinnati, OH, United States; ^2^Center for Psychedelic Drug Research and Education, The Ohio State University, Columbus, OH, United States; ^3^Humanities Program, University of Puget Sound, Tacoma, WA, United States

**Keywords:** psychedelic literature, poetry, poetics, consciousness studies, nonduality, William Blake, Aldous Huxley, Alan Watts

Literary studies has seen a recent explosion of interest in several fields that overlap with psychedelic studies. One is “cognitive literary studies,” which relates developments in neuroscience and psychology to the interpretation of literary texts. Others include the medical or health humanities and its subfield, narrative medicine, which explore the importance of storytelling and sense-making for the wellbeing of individuals and their communities. Despite sharing these similar methodologies and theoretical interests, literary studies remains underrepresented in the field of psychedelic studies and its associated conferences and publications. Regardless of this latency—which has no doubt been influenced by underfunding and deprioritization of the study of literature more generally—poetics are crucial to mapping the phenomenology of psychedelic experiences, and a proliferation of literary experiments could offer new ways of communicating the ineffable.

A key reason for this neglect is that research in the sciences and social sciences commonly assumes ontological positions that are fundamentally incompatible with psychedelic states as subjectively experienced. These positions include the commitment to materialism (reality is entirely reducible to a material substrate), physicalism (everything that exists is physical and dependent on mechanistic, natural laws) and scientism (the hard sciences provide the only genuine knowledge about reality). Even when studying the phenomena of conscious experience, many researchers presuppose that matter at the subatomic level constitutes the ground of reality and exists independently of human consciousness, that time and space as the necessary conditions of experience exist independently of the mind, and that consciousness is ultimately reducible to the material structure and processes of the brain. Guided by these widespread and closely-held assumptions, science dedicates itself to understanding the behavior of the material world—a world of matter and energy situated within time and space that unfolds according to established causal principles—while the social sciences commonly explore human behavior within the ontological framework created by these assumptions. Consequently, the discursive practices used by scientific and social scientific disciplines prioritize denotative language that suits the methods of empirical observation, mathematical logic, and the imperative for objectivity.

As Alan Watts explores in *The Joyous Cosmology*, the English language emphasizes “contrast and classification” in its denotative uses, which subtly reinforces the notion that reality is reducible to singular, isolated, material parts (Watts, 2013, p. 50). Although this view of reality often seems inevitable and intuitive in ordinary states of consciousness, psychedelic experiences frequently catapult even staunch materialists into a perspective where reality is seen as a dynamic play of eternal energy wherein the usual categories of opposition—self/other, subject/object, mind/matter—dissolve into awareness of interrelated unity. Given the persuasiveness and frequency of these sorts of experiences under the influence of psychedelics, it is unsurprising that a recent paper correlates psychedelic use with shifts away from “hard materialist” metaphysical beliefs (Timmermann et al., 2021). These experiences are thus ill-suited to the conventions of denotative language. To the extent that they are susceptible to description, they require highly figurative, connotative language rich in metaphor and symbolic meaning, which is the domain of poetry and other verbal, pictorial, and musical arts.

The most successful literary and artistic renderings of psychedelic states work not by providing reductive testimonies, but rather by disrupting our habitual patterns of cognition with an aesthetic that *enacts* the very experience it represents. Within psychedelic culture, the works of William Blake (1757–1827) include paradigmatic examples of literary art that seeks to transform consciousness by wrenching it from the lethargy of custom. Blake's expertise in rendering this poetics of disruption explains the deep impression he left—following Aldous Huxley's *The Doors of Perception*, which draws its title from Blake—on Beat Generation poets as well as writers, musicians, and artists of the 1960s psychedelic counterculture. Although present-day psychedelic researchers still allude to William Blake as a reference for describing psychedelic effects (Nutt, 2021), few scholars within the contemporary research revival have investigated the reasons for Blake's artistic and literary appeal within psychedelia (for an early exception, see Boon, 2002).

Although Blake knew nothing of psychedelics, his artistic vision was rooted in an anti-materialist conviction that “Mental Things are alone Real[;] what is Call[e]d Corporeal Nobody Knows of its Dwelling Place[; it] is in Fallacy & its Existence an Imposture” (Blake, 1997, p. 565). In its alignment with the premise of metaphysical idealism—i.e., that reality is grounded in mind, not in matter—Blake's claim is congruent with recurring components of psychedelic phenomenology, wherein the material world is commonly seen to be the manifestation of mind rather than its cause. Blake's art thus attempts to draw its readers into a reality as foreign to the materialist zeitgeist of his times as the psychedelic explorer's experience is to the scientific norms of ours—a reality experienced as infinite consciousness unfolding in boundless modes of expression. His term for this engine of expression is “Poetic Genius,” which draws upon *poiesis* (Greek for “a making”) and the Latin *genero* (to beget, produce). For Blake, “the Poetic Genius is the true Man, and … the body or outward form of Man is derived from the Poetic Genius” (Blake, 1997, p. 1). Consequently, when Blake writes that his literary art intends to “rouse the faculties to act,” his aim is to awaken the Poetic Genius in his readers so that they may become aware that their perceptions are actively creative rather than passive representations of sensory data emitted from a world of supposedly material objects independent of consciousness. Thus the creative imagination, regarded with suspicion by Enlightenment rationalists for producing pleasing but meaningless chimeras, is for Blake the activity of the individual mind participating in the generative processes of universal consciousness that render the natural world: “But to the Eyes of the Man of Imagination Nature is Imagination itself. As a Man is So he Sees” (Blake, 1997, p. 702).

To achieve this transformation of awareness in his readers, Blake employs what W. J. T. Mitchell terms a “composite art,” with an aesthetic that hovers in the ambiguous interplay between its verbal and visual elements (Mitchell, 2019). The verbal elements present a world of *process* whose only principle of permanence resides in the creative imagination that drives the process, while the illustrations—which reject literal representation for suggestive symbolism—underscore the complete degree to which the visual world behaves according to the activities of Poetic Genius, whether in its transpersonal or individual expression. The composite effect thus aims to disrupt the culturally entrenched belief in the primacy of the material world by drawing our awareness to the processes by which consciousness—acting through the Poetic Genius of individuals as localizations of what Huxley calls “Mind at Large” (Huxley, 2009, pp. 22–24)—constitutes the ground of reality itself. It is important to note that this perspectival shift does not depend on any predetermined ontological commitments from the reader/experiencer; rather, psychedelics and psychedelic poetics alike provide an impetus by which one can experience the world beyond the hegemonic and routinized confines of materialist assumptions.

Plate 14 from *The Marriage of Heaven and Hell* (1790) serves as a case study on Blake's approach to shifting reality frames through a poetics of disruption. Its famous line—“If the doors of perception were cleansed everything would appear to man as it is: infinite,” which inspired both the title of Huxley's *The Doors of Perception* and the name of the psychedelic rock band “The Doors”—exemplifies the aim of his artistic enterprise to disrupt our habitual cognitive filters so that we may better understand our *a priori* identity in consciousness (Blake, 1997, p. 39). An illustration sits at the top of the plate depicting a human figure with arms outstretched who appears to be flying directly at the reader. This figure hovers over and is partly immersed in flames that separate it from a stiffly prone, corpse-like human figure lying at right angles to the orientation of its airborne opposite. Beneath the illustration, the speaker declares: “The ancient tradition that the world will be consumed in fire at the end of 6000 years is true, as I have heard from Hell” (Blake, 1997, p. 39). Rather than suggesting sympathy with “Hell” as traditionally conceived, other plates treat flames as the element of transformation, and thus as a symbol of creative imagination. This symbolism informs the reader's interpretation of the speaker's proclamation: “For the cherub with his flaming sword is hereby commanded to leave his guard at the tree of life, and when he does, the whole creation will appear infinite and holy, whereas it now appears finite and corrupt” (Blake, 1997, p. 39). This proclamation implies that the foretold Biblical apocalypse is to be understood as an epiphany—ever available to the Poetic Genius—that the world is not susceptible to destruction, for it is not ultimately material in nature. Rather, for Blake, the ontological basis of the world *is* mind, and the mind is not bound by the limits of time and space, nor by the laws of causality that govern the world of material appearance.

Blake does not attempt to communicate this disruptive perspective through logical reasoning rendered in denotative language. Instead, such revelation comes about by “an improvement of sensual enjoyment” through the aesthetic of this very plate, which enacts the transformation it describes. This transformation invites readers to expunge “the notion that man has a body distinct from his soul,” and realize that all bodies—indeed, all material appearances—are the playful activities of mind projected through the senses (Blake, 1997, p. 39). The speaker then describes how they will bring about this shift in perspective—“by printing in the infernal method, by corrosives, which in Hell [i.e., the domain of fire as creative energy] are salutary and medicinal, melting apparent surfaces away and displaying the infinite which was hid” (Blake, 1997, p. 39). The speaker here refers to Blake's method of relief printing, which reveals inherent form through a process of eliminating the dross that obscures it. The plate thus anticipates Marshall McLuhan's assertion that “the medium is the message,” since its use of acidic corrosives in the “cleansing” process allows the plate's verbal and visual images to emerge from their bondage within the blank copper plate (McLuhan, 1994, p. 7). The images revealed through this process encourage readers to perform a similar act of liberation—one that melts away the static dualisms of picture/verse, body/mind, self/other, space/time to reveal consciousness as dynamically creative, infinite, and eternal. By dissolving these boundaries, Blake's creative works enact the kind of paradigm shift away from entrenched beliefs that is commonly attributed to psychedelics.

The therapeutic effects of psychedelics are similarly associated with their ability to change one's self-understanding by clearing attachments, assumptions, and limiting habits of mind. Using Blake's artistry as an example, we propose that psychedelic literary studies can contribute to psychedelic studies in several key ways. To the extent that psychedelics are regarded as “experiential medicines,” creative writers—whose trade is in the adept use of figurative language—are well-positioned to enact such transformative (and potentially therapeutic) experiences in their readers. These poetic strategies provide insight into the processes by which psychedelics disrupt conventional frameworks and suggest new experiential paradigms. Psychedelic users (including Huxley) have recounted how ideas that previously seemed preposterous or nonsensical suddenly became meaningful and coherent in a state of heightened affect and aesthetic sensitivity (Huxley, 2009, pp. 18–19). By replicating the feelings associated with such experiences, poetry as a technology can convey psychedelic perspectives in a manner that exceeds the capabilities of scientific discourse and method.

Blake's liberatory aesthetic thus offers a paradigmatic example of the ways in which literary studies specifically, and the arts more generally, can invite readers into modes of experience that challenge our standard conceptual frameworks for understanding ourselves and the world. Even when attempted renderings of psychedelic states are incomplete or imperfect, each new metaphor potentially functions as a useful tool for navigating and communicating future experiences, as Richard Doyle suggests in *Darwin's Pharmacy* (Doyle, 2011). Since the poetic creation of metaphors and other types of figurative language shapes the contents and interpretations of psychedelic experiences, poetry has been foundational to our understanding of their nature. As a result, poetry will continue to influence the future of psychedelic science, whether or not that influence is acknowledged or institutionally supported.

## Author contributions

ND and GE contributed to the drafting and editing of this manuscript. Both authors contributed to the article and approved the submitted version.
